# Serum versus synovial fluid interleukin-6 for periprosthetic joint infection diagnosis: a systematic review and meta-analysis of 30 diagnostic test accuracy studies

**DOI:** 10.1186/s13018-022-03458-x

**Published:** 2022-12-24

**Authors:** Jian Li, Qian Zhou, Biquan Deng

**Affiliations:** 1 Department of Orthopaedics, The Central Hospital of Wuhan, Tongji Medical College, Huazhong University of Science and Technology, Wuhan, 430014 China; 2 Department of Pharmacy, The Central Hospital of Wuhan, Tongji Medical College, Huazhong University of Science and Technology, Wuhan, 430014 China

**Keywords:** Knee, Hip, Arthroplasty, Interleukin-6, Diagnostic meta-analysis

## Abstract

**Background:**

Early and accurate detection of periprosthetic joint infection (PJI) after hip and/or knee arthroplasty remains challenging. This systematic review and meta-analysis of diagnostic test accuracy studies aimed to evaluate the diagnostic accuracy of serum and synovial fluid interleukin (IL)-6 in detecting PJI.

**Methods:**

We searched 3 databases for studies through December 31, 2021, using medical sub-headings terms and keywords. Studies reported sensitivity and specificity of serum and synovial fluid IL-6 in detecting PJI were considered. We calculated the pooled sensitivity, specificity, positive and negative likelihood ratio, diagnostic odds ratio (DOR), and the area under the summary receiver operating characteristic curve (AUC) to evaluate the diagnostic accuracy of serum and synovial fluid IL-6.

**Results:**

Thirty studies were included. The pooled sensitivity, specificity, positive and negative likelihood ratio, DOR, and AUC of serum IL-6 in detecting PJI were 0.76 (0.69–0.81), 0.88 (0.82–0.92), 6.2 (4.3–9.0), 0.28 (0.22–0.35), 22 (14–36), and 0.88 (0.85–0.91), respectively. However, synovial fluid IL-6 achieved a pooled sensitivity of 0.87 (0.75–0.93), specificity of 0.90 (0.85–0.93), positive and negative likelihood ratio of 8.5 (5.3–13.6) and 0.15 (0.08–0.29), DOR of 57 (21–156), and AUC of 0.94 (0.92–0.96), which were higher than serum IL-6.

**Conclusions:**

Synovial fluid IL-6 test may be a promising test for PJI after hip and/or knee arthroplasty. However, considering the limited volume of synovial fluid and invasive acquisition of synovial fluid IL-6, serum IL-6 test may be also considered.

**Supplementary Information:**

The online version contains supplementary material available at 10.1186/s13018-022-03458-x.

## Introduction

As a serious complication after knee and hip arthroplasties, periprosthetic joint infection (PJI) has been regarded as the main contributor to joint arthroplasty failure (20.4%) [1] and revision after joint arthroplasty [2]. Studies revealed that the number of patients who received arthroplasty resulted from PJI is five times more than patients who did not receive an arthroplasty for a PJI [3, 4]. PJI was also associated with an increased morbidity and mortality rate, and it therefore significantly increased the economic burden on the healthcare system [5, 6]. Early and accurate detection of PJI after knee and hip arthroplasty has become an important approach to minimize the risk caused by PJI. Unfortunately, it remains a challenge for early and accurately detecting PJI due to optimal diagnostic method is not available [7].

The current standard for the diagnosis of prosthetic joint infection is diagnostic criteria, and a series of tests have been offered to comprehensively detect PJI hip and/or knee arthroplasty [8]. It is noted that diagnostic tests based on biomarkers were found to have good diagnostic value in the early and accurate detection of PJI hip and/or knee arthroplasty in recent years, such as C-reactive protein (CRP) [9], α-defensin [10], leukocyte esterase [11], and interleukin-6 (IL-6) [12], and these biomarkers are a component of the diagnostic criteria. Nevertheless, meta-analysis indicated an inadequate overall diagnostic accuracy [13]. Among the existing biomarkers, the role of IL-6 in detecting PJI has been extensively investigated, and the clinical significance of IL-6 in distinguishing between infected and aseptic failed total joint replacements has also been suggested [14].

Currently, two meta-analyses [15, 16] have evaluated the diagnostic accuracy of IL-6 in detecting PJI and indicated the excellent diagnostic value of IL-6 in detecting PJI. However, different sources of IL-6 were speculated to be associated with different diagnostic accuracies; a previous meta-analysis was therefore conducted to determine the difference between serum and synovial fluid IL-6 in detecting PJI after hip, knee, and/or shoulder replacement. However, it is unclear whether these findings were suitable to patients only receiving hip and/or knee replacements. Moreover, numerous studies continued to focus attention on this issue after previous meta-analysis because a definitive conclusion as not yet been achieved for specific population. We therefore performed this systematic review and meta-analysis of diagnostic test accuracy (DTA) studies to further evaluate the diagnostic accuracy of serum and synovial fluid IL-6 in detecting of PJI after hip and/or knee arthroplasty through combining available studies.

## Materials and methods

We performed this diagnostic meta-analysis according to the methods recommended by the Cochrane handbook [17] and reported it in line with the Preferred Reporting Items for Systematic Reviews and Meta-Analyses (PRISMA) extension for Diagnostic Test Accuracy (DTA) [18]. No ethical approval and patient’s informed consent was required because this is a meta-analysis of previously published studies. We did not register the formal protocol in any public platform.

### Study retrieval

We systematically searched 3 databases including PubMed, EMBASE, and the Cochrane library for identifying relevant studies from their inception through to 31 December, 2021. Study search was performed by two qualified independent investigators (** and **). We used the following terms and its analogs to develop search query with Boolean operators: “periprosthetic joint infection”, “interleukin-6”, and “joint prosthesis”. The sensitivity of the search query was modified according to the unique requirements of each database. Additionally, we screened reference lists of eligible studies and published reviews to avoid missing relevant studies. No restriction on publication language and status was applied to literature retrieval. The third senior investigator (**) was consulted for resolving any disagreements during study retrieval. Details of the search query are summarized in Additional file 1: Table S1.

**Table 1:** Basic characteristics of 30 studies included in this diagnostic meta-analysis

Study	Country	Design	Sample (PJI/Aseptic)	Male/Female	Mean age, years (PJI vs Aseptic)	Affected part	Exclusion of inflammatory diseases	Diagnosis of PJI	Cut-off, pg/mL	Type of PJI
serum IL-6										
Abou, et al., [25]	Egypt	PS	40 (11/29)	21/19	58.4	Mixed	Yes	MSIS	10.4	No clear information
Ackmann, et al., [26]	Germany	RS	119 (52/67)	69/50	70.5 vs 68	Mixed	Yes	MSIS	10.0	Chronic PJI
Buttaro, et al., [28]	Argentina	PS	69 (11/58)	50/19	68	Hip	Yes	Other	10.0	No clear information
Di, et al., [32]	USA	PS	58 (17/41)	25/33	63	Mixed	Yes	Other	10.0	Chronic PJI
Glehr, et al., [38]	Austria	PS	84 (55/29)	38/46	NA	Mixed	Yes	MSIS	4.7	No clear information
Yu, et al., [49]	China	RS	121 (20/101)	35/86	68.2 vs 64.9	Mixed	Yes	MSIS	8.1	Acute PJI
Yu, et al., [50]	China	RS	139 (62/77)	46/93	65.85 vs 64.60	Mixed	No	MSIS	8.980	No clear information
Chu, et al., [29]	China	RS	35 (16/19)	14/21	66 vs 65	Mixed	NA	MSIS	NA	No clear information
Klim, et al., [41]	Austria	PS	84 (55/29)	38/46	65.7 vs 65.1	Mixed	Yes	MSIS	5.7	No clear information
Grzelecki, et al., [40]	Poland	PS	85 (45/40)	25/60	65.5 vs 68.3	Mixed	Yes	Other	7	Chronic PJI
Xu, et al., 2019	China	RS	318 (129/189)	NA	NA	Mixed	Yes	MSIS	8.57	No clear information
Yin, et al., [48]	China	NA	35 (15/20)	21/14	66.4 vs 68.7	Mixed	NA	MSIS	23.05	No clear information
Erdemli, et al., [34]	Turkey	PS	88 (36/25)	27/61	68.7	Mixed	No	MSIS	16.2	No clear information
Tang, et al., [46]	China	RS	52 (21/31)	22/30	65.5 vs 61.7	Mixed	No	MSIS	7.5	No clear information
Worthington, et al., [52]	UK	PS	46 (16/30)	25/21	72	Hip	Yes	NA	9	No clear information
Bottner, et al., [27]	USA	PS	78 (21/57)	41/37	63.5 vs 64.8	Mixed	No	Other	12.0	No clear information
Elgeidi, et al., [33]	Egypt	PS	40 (11/29)	21/19	59.6 vs 57.9	Mixed	Yes	Other	10.4	No clear information
Qu, et al., [12]	China	RS	86 (16/70)	48/38	56 vs 54	Hip	No	MSIS	8.12	No clear information
Qin, et al., [44]	China	PS	93 (37/56)	58/35	74.57 vs 72.15	Mixed	Yes	MSIS	6.7	No clear information
Qin, et al., [45]	China	PS	70 (35/35)	35/35	63.66 vs 66.2	Mixed	Yes	MSIS	6.1	No clear information
Gollwitzer, et al., [39]	Germany	PS	35 (15/20)	12/23	71 vs 70	Mixed	Yes	Other	1.89	Chronic PJI
Randau, et al., [14]	Germany	PS	120 (48/72)	47/73	NA	Mixed	NA	MSIS	2.6	No clear information
Randau, et al., [14]	Germany	PS	120 (48/72)	47/73	NA	Mixed	NA	MSIS	6.6	No clear information
synovial IL-6										
Sharma, et al., [51]	USA	NA	107 (50/57)	57/50	65.6 vs 66.2	Mixed	NA	MSIS	NA	Acute, chronic, and hematogenous PJI
Fröschen, et al., [37]	Germany	RS	32 (14/18)	11/21	73.8 vs 62.28	Mixed	No	MSIS	1975	No clear information
Ettinger, et al., [35]	Germany	PS	72 (12/60)	NA	NA	Mixed	No	MSIS	373	Chronic PJI
Mihalič, et al., [42]	Slovenia	PS	48 (11/37)	23/25	68	Mixed	No	Other	2300	Acute and chronic PJI
Frangiamore, et al., [36]	USA	PS	90 (31/59)	39/51	63 vs 65	Mixed	No	MSIS	8671.0	No clear information
Deirmengian, et al., [31]	USA	PS	95 (29/66)	44/51	67 vs 66	Mixed	No	MSIS	2300	No clear information
Nilsdotter-Augustinsson, et al., [43]	Sweden	PS	131 (25/106)	64/67	NA	Hip	No	Other	10,000	No clear information
Deirmengian, et al., [30]	USA	PS	51 (14/37)	23/28	65	Mixed	No	Other	13,350	No clear information
Yu, et al., [50]	China	RS	139 (62/77)	46/93	65.85 vs 64.60	Mixed	No	MSIS	6590.289	No clear information
Qin, et al., [44]	China	PS	93 (37/56)	58/35	74.57 vs 72.15	Mixed	Yes	MSIS	1855.36	Chronic PJI
Qin, et al., [45]	China	PS	70 (35/35)	35/35	63.66 vs 66.2	Mixed	Yes	MSIS	1950	Chronic PJI
Gollwitzer, et al., [39]	Germany	PS	35 (15/20)	12/23	71 vs 70	Mixed	Yes	Other	1896.56	No clear information
Randau, et al., [14]	Germany	PS	120 (48/72)	47/73	NA	Mixed	No	MSIS	2100	No clear information
Randau, et al., [14]	Germany	PS	120 (48/72)	47/73	NA	Mixed	No	MSIS	9000	No clear information

*PJI* Periprosthetic joint infection, *IL-6* Interleukin-6, *PS* Prospective study, *RS* Retrospective study, *MSIS* Musculoskeletal Infection Society criteria, *NA* Not available

### Selection criteria

We selected eligible studies according to the following criteria [16, 19]: (a) eligible patients were identified with PJI with recognized diagnostic criteria after hip and/or knee replacement; (b) serum or synovial fluid sample was obtained for diagnostic investigation in eligible studies; (c) studies reported the data of true positives (TP), false positives (FP), false negatives (FN), true negative (TN) or the sensitivity and specificity. We excluded ineligible studies according to the following criteria: (a) studies were performed to investigate the diagnostic value of IL-6 in detecting PJI after shoulder and/or elbow replacement; (b) ineligible study design such as narrative review, animal studies, or case report; (c) no control group was designed or direct comparison between samples were not available; (d) repeated reports focusing on the same topic published by the same group but with insufficient data and relatively poor quality; and (e) essential data were not accessible in original studies and additional data could not be added through contacting the leading author.

### Study selection

Two independent qualified investigators selected eligible studies based on the selection criteria as follows: (a) all records identified from 3 electronic databases were imported into EndNote X9 to build literature database; (b) after removal of duplicate records, the titles and abstracts of retained records were screened; and (c) eligibility of the remaining studies was evaluated finally based on the screening for full-text. The third senior investigator was consulted for resolving any disagreements during study selection.

### Data extraction

Two independent qualified investigators extracted the essential data from original studies, including the name of the first author, publication year, country, study design, the number of patients included for the final analysis, the number of PJI and aseptic cases, the diagnostic criteria of PJI, and the part of infected joint (knee, hip or mixed parts), cut-off value, and the diagnosis result including the numbers of TP, FP, FN, and TN, or sensitivity and specificity. We contacted the leading author to collect essential data if necessary. The third senior investigator was consulted for resolving any disagreement during data extraction.

### Quality assessment

Two independent qualified investigators (** and **) evaluated the risk of bias and concerns about applicability of the included studies using the Quality Assessment for Studies of Diagnostic Accuracy Score (QUADAS) tool [20]. This tool determined the methodological quality of each study from four domains, including patient selection, index test, reference standard, and flow and timing. We assessed the risk of bias for all domains and applicability for the first three domains. We rated each domain as “low,” “unclear,” and “high” risk. The third senior investigator (**) was consulted for resolving any disagreement about quality assessment.

### Statistical analysis

We firstly calculated the TP, FP, FN, and TN based on the available information extracted from the original studies, quantitative indicators with corresponding 95% confidence interval (CI) were then estimated for evaluating the diagnostic value of the serum and synovial fluid IL-6 for the detection of PJI after knee or hip arthroplasty, including the pooled sensitivity and specificity, the positive likelihood ratio, negative likelihood ratio, diagnostic odds ratio (DOR), and the area under the summary receiver operating characteristic (SROC) curve (AUC) [21, 22]. Meanwhile, we evaluated statistical heterogeneity across studies based on the *χ*^2^ test and *I*^*2*^ statistics, and *I*^*2*^ ≥ 50% suggested the presence of substantial heterogeneity [23]. Nevertheless, we used the random-effects model to perform data synthesis because variations between studies should not be ignored in real settings. Additionally, we performed the subgroup analysis to furtherly investigate the influence of various characteristics on the diagnostic accuracy of serum and synovial fluid IL-6 test for the diagnosis of PJI. Finally, we created the Deek’s funnel plot to evaluate the risk of publication bias [24]. Data analysis was performed by using STATA 14.0 (StataCorp, College Station, TX, USA) with the “midas” module.

## Results

### Literature retrieval

**Fig. 1:**
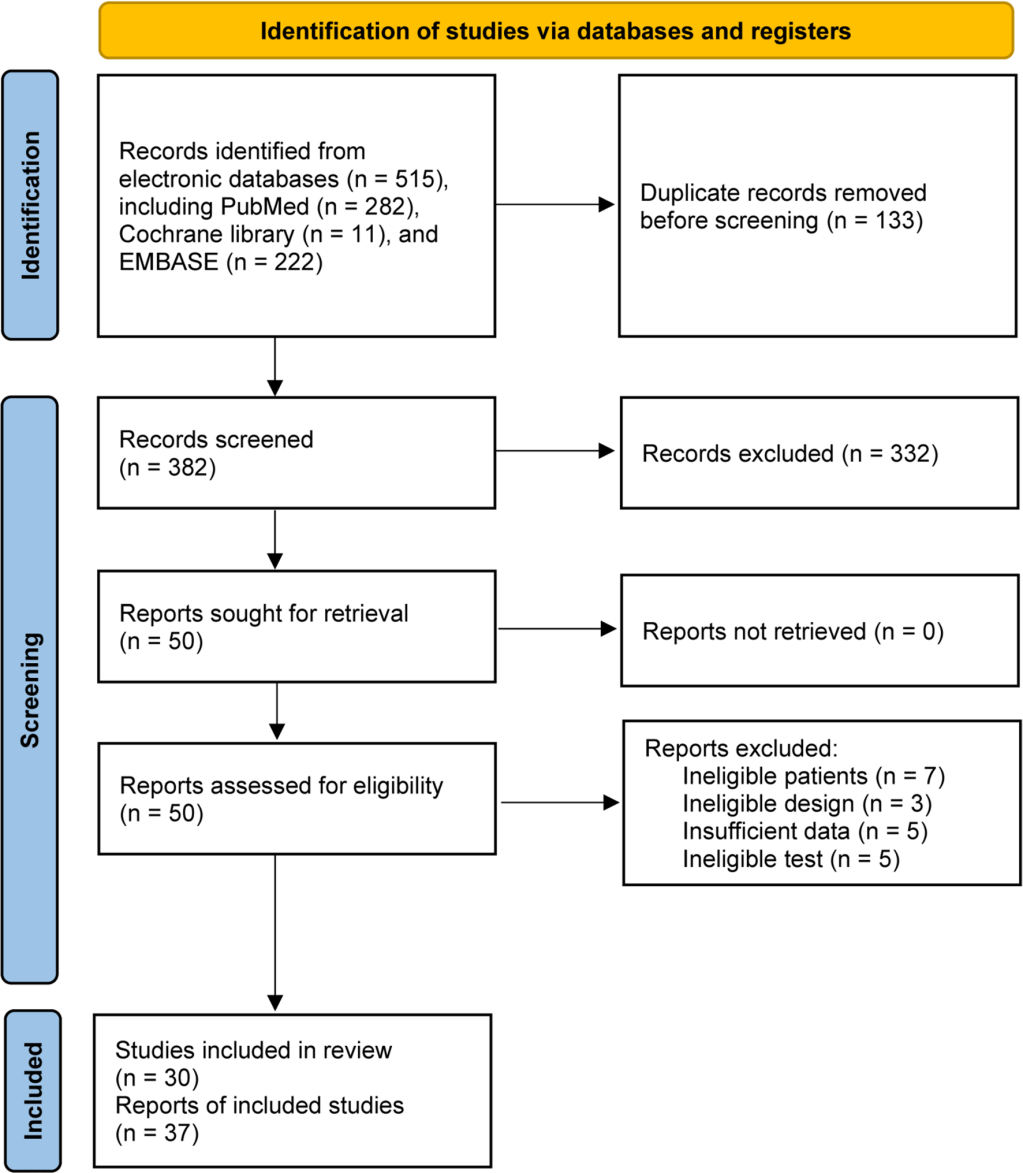
The PRISMA flowchart of study selection

We identified 515 records from 3 databases. A total of 133 duplicate records were removed using EndNote software. We excluded 332 ineligible studies after screening the titles and abstracts. Among 50 studies retained for further eligibility evaluation, 20 studies were excluded due to ineligible participants (*n* = 7), ineligible study design (*n* = 3), insufficient data (*n* = 5), and ineligible test (n = 5). Finally, 30 studies[12, 14, 25–45] were judged for meeting selection criteria, including 23 reports for serum IL-6 and 14 reports for synovial fluid IL-6. The flow diagram of the study selection is displayed in Fig. 1.

### Basic characteristics of eligible studies

**Fig. 2:**
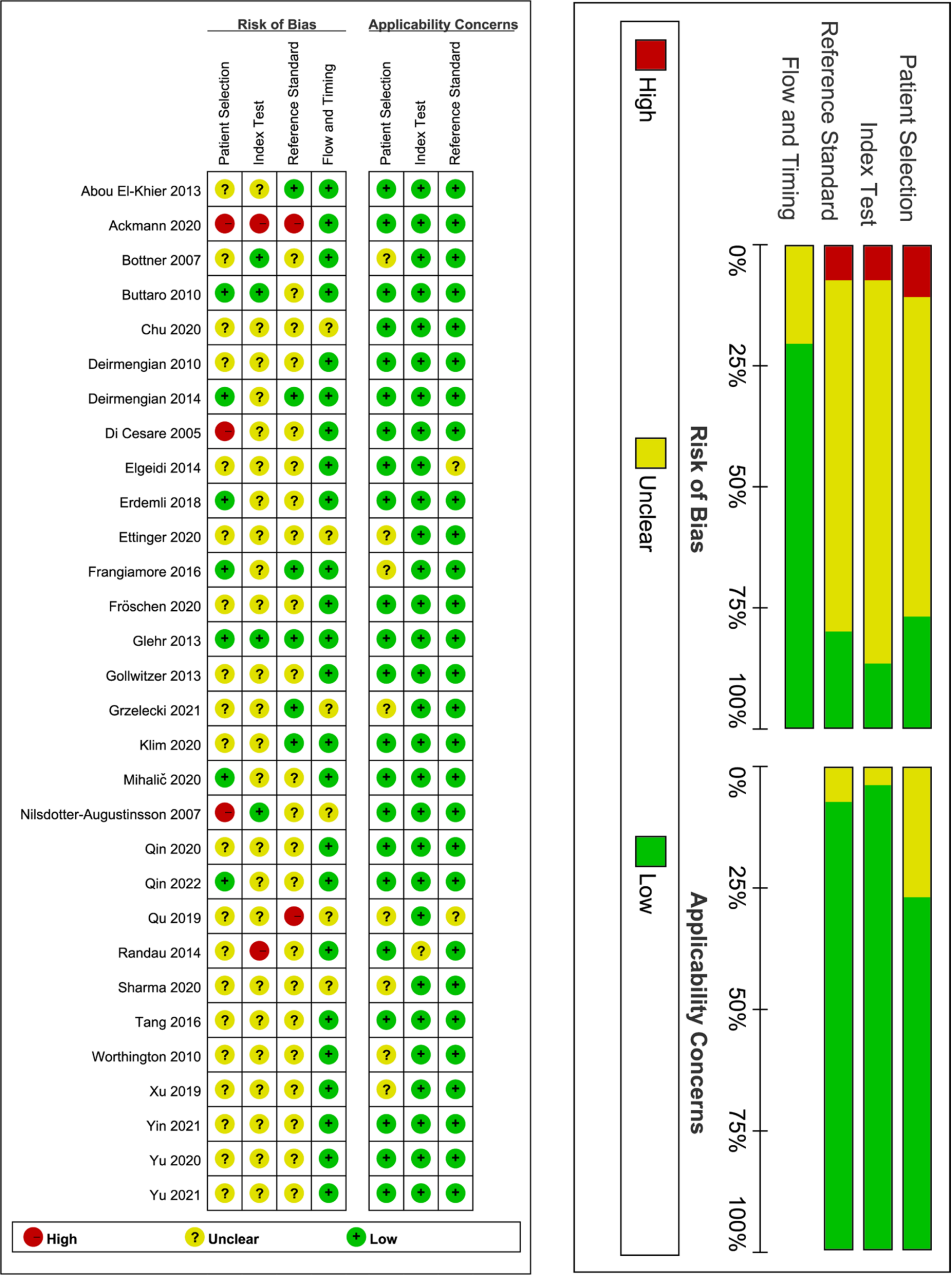
Quality assessment of the included studies

We designed Table 1 to summarize the basic characteristics of 30 eligible studies. Totally, 3218 patients were accumulated, involving 1223 patients with PJI and 1995 patients with aseptic loosing. All studies were published between 2005 and 2021. Among the included studies, 9 studies [12, 29, 44–50] performed in China, 7 studies [27, 30–32, 36, 51] in USA, 5 studies [14, 26, 35, 37, 39] in Germany, 2 studies [25, 33] in Egypt, 2 studies [38, 41] in Austria, and remaining studies in Argentina [28], Poland [40], Turkey [34], UK [52], Slovenia [42], and Sweden [43], respectively. Four studies [12, 28, 43, 52] enrolled patients receiving hip replacement, but remaining studies [14, 25–27, 29–42, 44–49, 51, 52] included patients undergoing both hip and knee replacements. Twenty studies [12, 14, 25, 26, 29, 31, 34–38, 41, 44–51] clearly reported to use the Musculoskeletal Infection Society criteria (MSIS) criteria for the diagnosis of PJI. The methodological quality of included studies was moderate, which is shown in Fig. 2.

### Diagnostic accuracy of serum and synovial fluid IL-6

**Fig. 3:**
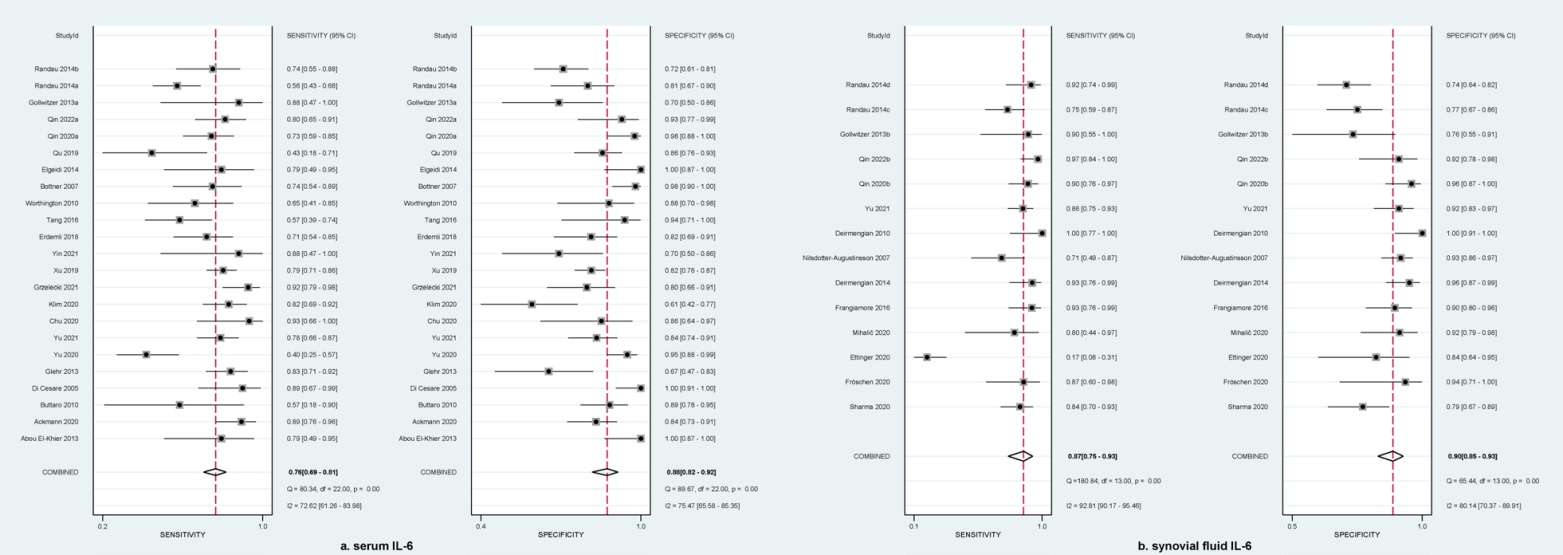
Forest plot of the pooled sensitivity and specificity of serum IL-6 **a** and synovial fluid IL-6 **b** for diagnosis of periprosthetic joint infection

**Fig. 4:**
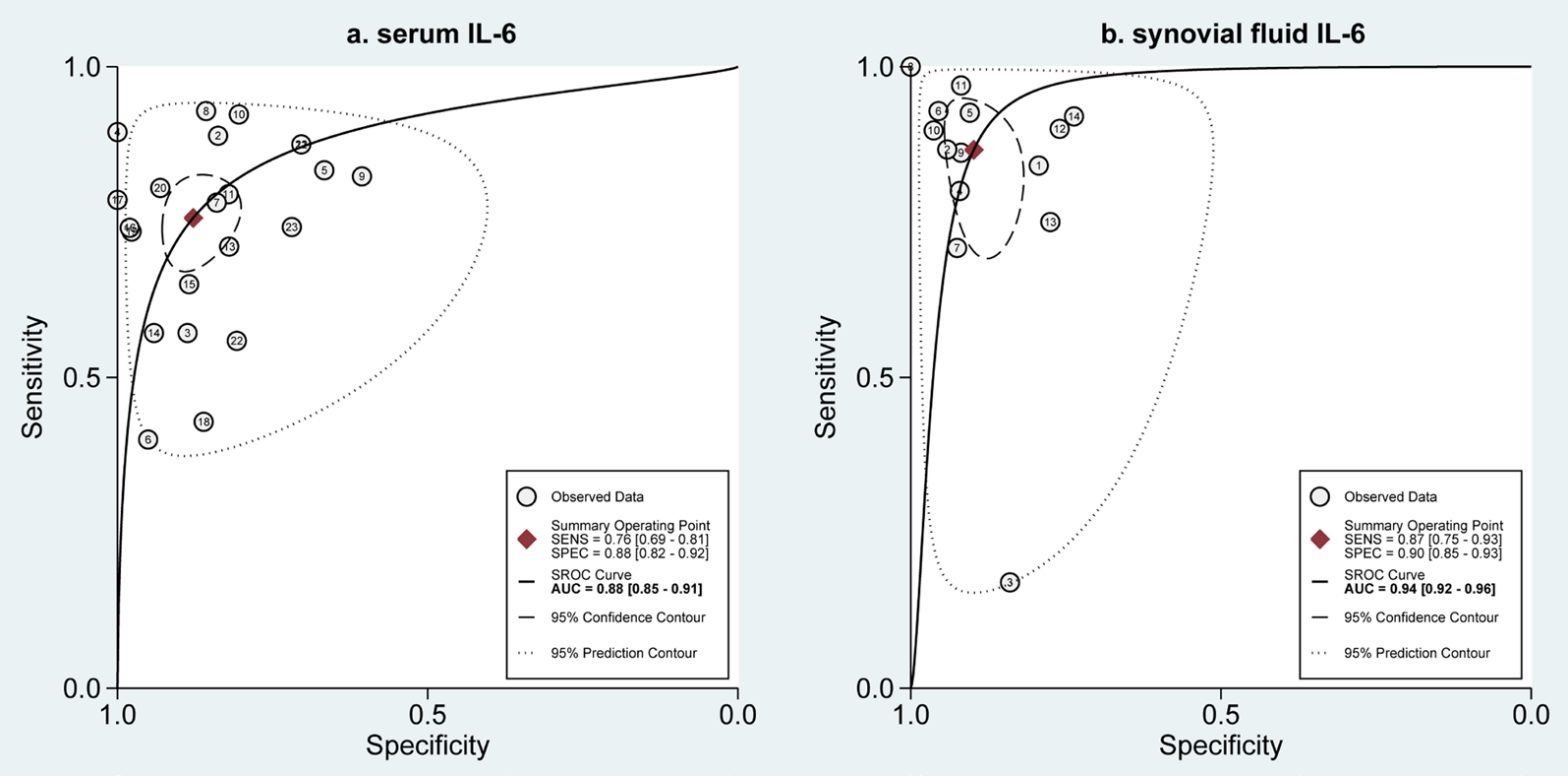
SROC curve of serum IL-6 **a** and synovial fluid IL-6 **b** for diagnosis of periprosthetic joint infection

A total of 23 reports evaluated the diagnostic accuracy of serum IL-6 for detecting PJI after hip and/or knee replacement, and meta-analysis suggested that, as shown in Fig. 3a, the pooled sensitivity and specificity was 0.76 (95%CI 0.69–0.81) and 0.88 (95%CI 0.82–0.92), respectively. Meanwhile, serum IL-6 reached a relatively high diagnostic accuracy, with an AUC of 0.88 (95%CI 0.85–0.91), which is shown in Fig. 4a. For the evaluation of diagnostic accuracy of synovial fluid IL-6, 14 reports were provided for data analysis. Meta-analysis suggested that, as shown in Fig. 3b, the pooled sensitivity and specificity were 0.87 (95%CI 0.75–0.93) and 0.90 (95%CI 0.85–0.93), respectively. Additionally, serum IL-6 received a higher diagnostic accuracy, with an AUC of 0.94 (95%CI 0.92–0.96) (Fig. 4b). This indicated that the diagnostic performance of synovial fluid IL-6 for PJI was superior to serum IL-6.

### Evaluation of the clinical utility

**Fig. 5:**
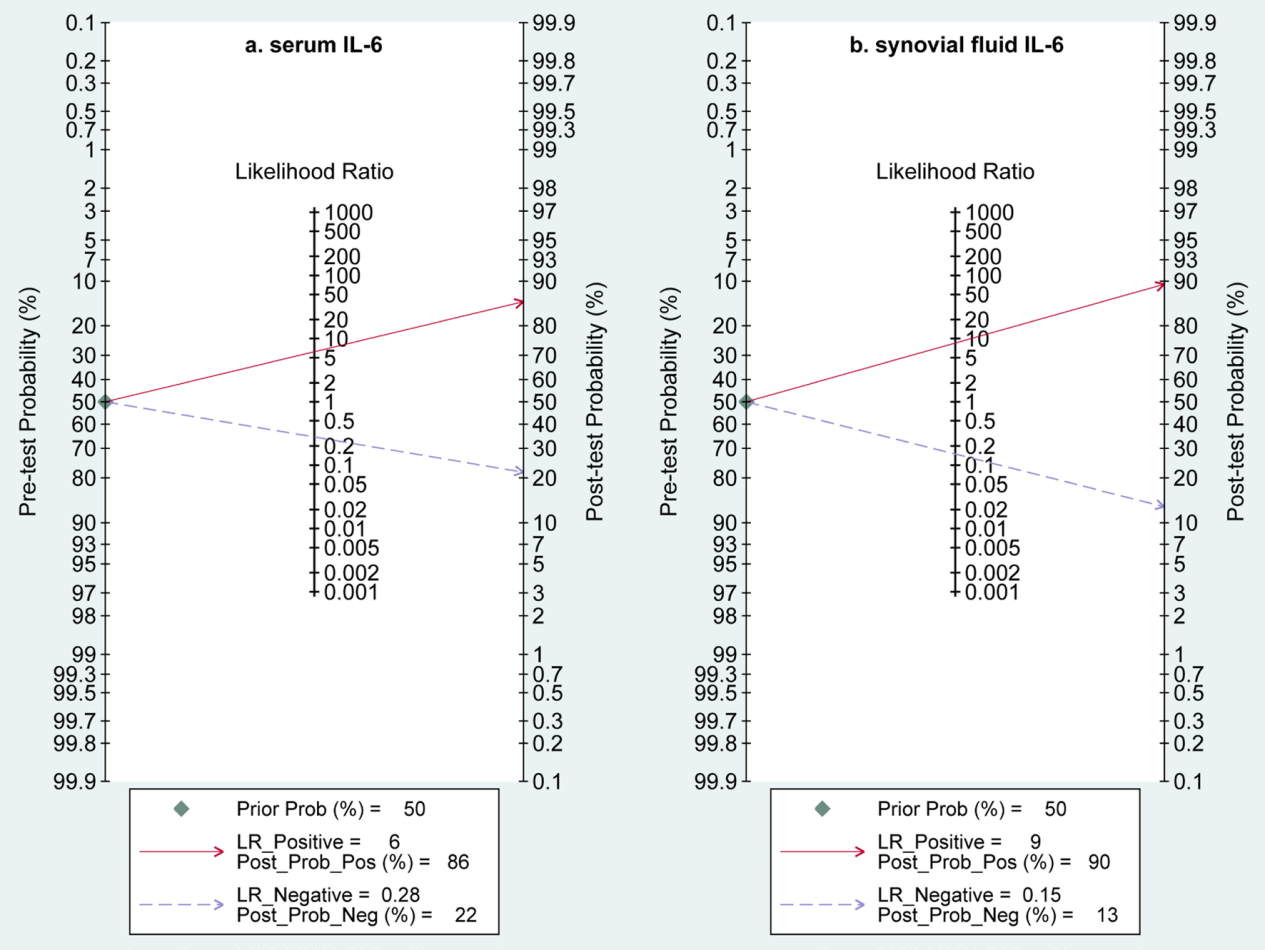
Fagan's nomogram of the post-test probability of IL-6 for diagnosis of periprosthetic joint infection based on the pre-test probability of 50% in serum IL-6 **a** and synovial fluid IL-6 **b**

Meta-analysis suggested that, as shown in Fig. 5, serum IL-6 achieved a positive likelihood ratio of 6.2 (95%CI 4.3–9.0) and a negative likelihood ratio of 0.28 (95%CI 0.22–0.35) for detecting PJI; however, synovial fluid IL-6 achieved a positive likelihood ratio of 8.5 (95%CI 5.3–13.6) and a negative likelihood ratio of 0.15 (95%CI 0.08–0.29). Moreover, the DOR of serum and synovial fluid IL-6 was 22 (95%CI 14–36) and 57 (95%CI 21–156), respectively. We designed 50% pre-test probabilities to estimate the post-test probability in this study, and a post-test probability of 22% was achieved for PJI in serum IL-6 test and 13% in synovial IL-6 test. This suggested synovial IL-6 test was linked to higher clinical utility compared to serum IL-6 test.

### Subgroup analysis

Substantial heterogeneity was observed for both serum and synovial fluid IL-6 tests. Subgroup analyses were therefore performed to check the robustness of pooled results based on pre-designed criteria, including exclusion of chronic inflammatory diseases or not, the part of affected joints (mixed joint replacements or hip replacement), the diagnostic criteria for PJI (MSIS criteria or others), study design (prospective or retrospective), the number of patients included for analysis (60 for serum IL-6 and 80 for synovial fluid IL-6), and the cut-off criteria (10 pg/ml for serum IL-6 and 2300 pg/ml for synovial fluid IL-6). As shown in Additional file 2: Table S2, subgroup analysis confirmed the robustness of pooled results. However, it is noted that the number of patients included for analysis and the cut-off criteria for PJI might have an impact on the diagnostic accuracy.

### Publication bias

**Fig. 6:**
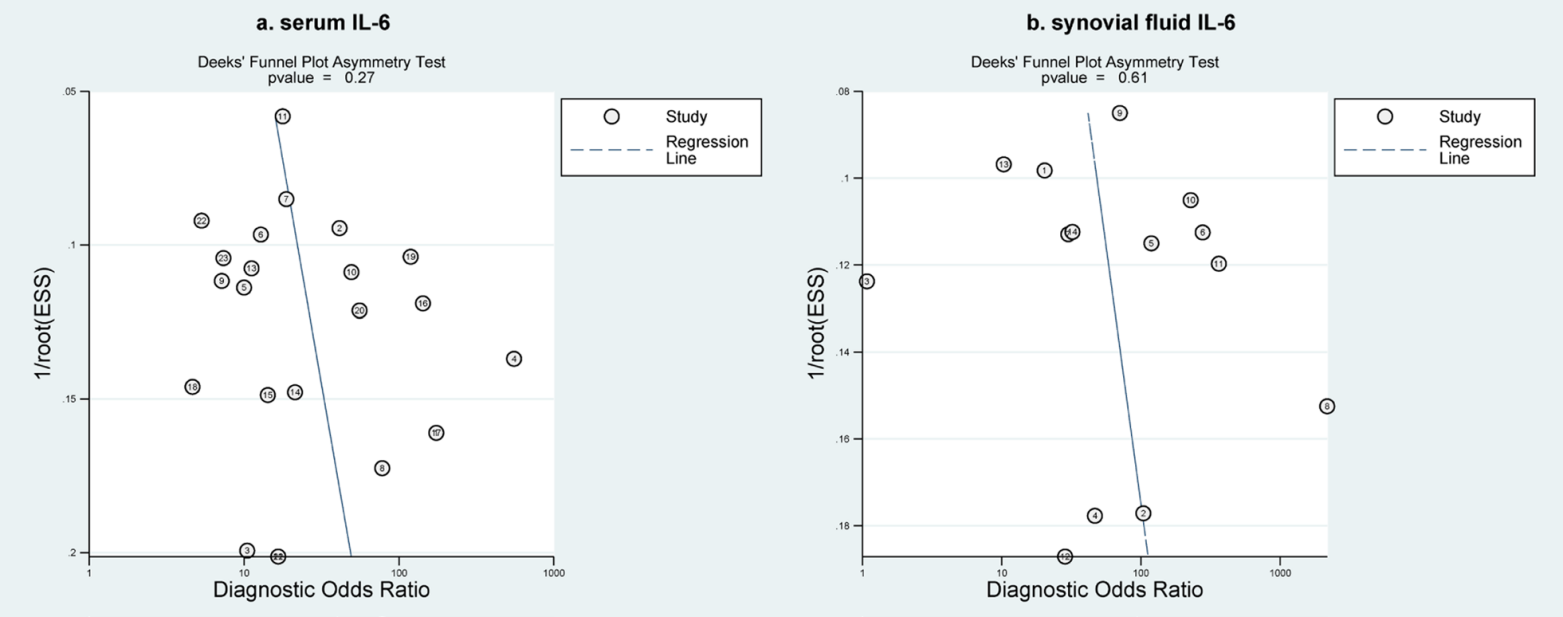
Deek's funnel plot of serum IL-6 **a** and synovial fluid IL-6 **b**

Deek’s funnel plot suggested that the studies were symmetrically located on both sides of the regression line. Furthermore, the asymmetric test for Deek’s plot quantitatively disclosed absence of publication bias for both serum and synovial fluid IL-6, with a P value of 0.27 and 0.61, respectively (Fig. 6).

## Discussion

Although IL-6 has been suggested to have relatively high diagnostic value in detecting PJI, and difference between serum and synovial fluid IL-6 tests in detecting PJI after hip, knee and shoulder replacements has also been initially evaluated, there was no systematic review and meta-analysis to evaluate the diagnostic accuracy of serum and synovial fluid IL-6 tests in detecting PJI among patients who underwent only hip and knee replacements. In this systematic review and meta-analysis of 30 DTA studies, we evaluated the diagnostic value of both serum and synovial fluid IL-6 tests for detecting PJI after hip and knee replacements, and found that synovial fluid IL-6 might be preferentially prescribed for detecting PJI after hip and knee replacements owing to its higher sensitivity, specificity, DOR, and diagnostic accuracy. Certainly, serum IL-6 might also be considered for detecting PJI after hip and knee owing to its comparable specificity to synovial fluid IL-6 because the volume of synovial fluid is limited and the acquisition of synovial fluid IL-6 is invasive. Moreover, more studies are required to further determine the optimal cut-off value of both serum and synovial fluid IL-6 tests because a negative association between diagnostic accuracy of IL-6 and cut-off criteria has been revealed.

Up to now, numerous systematic reviews and meta-analyses of DTA studies evaluated the diagnostic value of IL-6 in detecting PJI; however, a definitive conclusion has not yet been achieved due to several limitations, which were further explained in the following contents. In 2017, Lee et al. [13] found that synovial fluid IL-6 test was associated with higher diagnostic accuracy, indicating an AUC, sensitivity and specificity of 0.95, 0.81 and 0.94, respectively. In the same year, another meta-analysis reported a similar diagnostic accuracy, with an AUC of 0.956 [53]. It is noted that these findings were calculated from 5 [13] or 7 [53] eligible studies although they achieved consistent diagnostic accuracy with our meta-analysis; however, 14 reports were included in this study to generate more robust and reliable results. In 2020, a meta-analysis by Li et al. [54] found that serum IL-6 achieved a pooled sensitivity, specificity and DOR of 0.87, 0.83, and 36.27, respectively, as well as an AUC of 0.92. Insufficient eligible studies impaired the reliability of pooled results although a higher diagnostic accuracy was achieved compared with our study, in which 23 eligible reports were included for the estimation of diagnostic accuracy.

In 2017, Xie et al. performed a systematic review and meta-analysis of 17 DTA studies to investigate the relative diagnostic values of serum and synovial fluid IL-6 tests for PJI after hip, knee and shoulder replacements [19], suggesting that synovial fluid IL-6 test had a higher AUC (0.96 vs. 0.83) and DOR (101 vs. 20) as well as comparable specificity (0.90 vs. 0.89) relative to serum IL-6 test. Based on these results, authors concluded that serum IL-6 may be regularly prescribed for detecting PJI owing to its relatively high specificity although it had less sensitive than synovial fluid IL-6 test. Yoon et al. [15] included 16 eligible DTA studies for data analysis and found that IL-6 achieved an AUC of 0.93 in detecting PJI after hip, knee, shoulder, and/or elbow replacement, with a pooled sensitivity and specificity of 0.83 and 0.91, respectively. Meanwhile, Tian et al. performed a diagnostic meta-analysis, and pooled results suggested a higher diagnostic accuracy (0.91), corresponding to a sensitivity of 0.80 and a pooled specificity of 0.89 [16].

Compared with previous systematic reviews and meta-analyses of DTA studies, our study minimizes the variation in patient’s characteristics because only patients receiving hip and/or knee replacement were considered. Moreover, this was the first meta-analysis to determine the diagnostic value of serum and synovial fluid IL-6 in detecting PJI after hip and/or knee replacement through combining the greatest number of eligible studies. As an example, our study confirmed that synovial fluid IL-6 test was associated with higher DOR compared with serum IL-6 test, which was directionally consistent with previous meta-analysis. Specificity speaking, a pooled DOR of 57 with a corresponding 95%CI of 21 to 156 was generated in our study, while previous systematic reviews and meta-analyses of DTA studies reported a pooled DOR of 101, with a 95%CI of 28 to 358.

Our systematic review and meta-analysis of 30 DTA studies had some limitations. First, although we selected a random-effects model to calculate results, it is still a fact that substantial heterogeneity was present for both serum and synovial fluid IL-6 tests, which might lower the reliability of our findings. However, we believed that all findings from the present study could be preferentially considered in clinical practice because subgroup analysis confirmed the robustness of our results. Second the formal protocol of this systematic review and meta-analysis of DTA studies was not registered in any platform. However, we strictly performed data analysis and reported pooled results according to the recommended criteria, which greatly enhance the strictness and reliability of this study. Third, we included the greatest number of eligible studies to update the diagnostic accuracy of serum and synovial fluid IL-6 based on a systematic literature retrieval; however, the risk of missing eligible studies could not be avoided because other databases such as Web of Science and China National Knowledge Infrastructure (CNKI) were not retrieved. Fourth, we performed a series of subgroup analyses to investigate the contribution of some important factors to statistical heterogeneity; however, statistical heterogeneity was not significantly reduced after these subgroup analyses. Therefore, our findings should be interpreted with caution, as further analysis cannot be performed based on other potential factors. Finally, it must be acknowledged that distinguishing the diagnostic values of serum or synovial IL-6 for different types of PJI is important because chronicity of the PJI makes a different diagnosis criterion. However, most of the eligible studies did not provide details on PJI type, so further analysis by PJI type was not possible.

## Conclusion

Synovial fluid IL-6 test has significantly higher diagnostic accuracy for the detection of PJI among patients undergoing hip and/or knee replacement. Although serum IL-6 test is less sensitive than synovial fluid IL-6 test, it can also be considered for patients with prosthetic failure due to its comparable specificity to synovial fluid IL-6 test. Certainly, our findings should be interpreted with caution due to significant statistical heterogeneity. Moreover, more studies should be performed to determine the optimal cut-off value because it has a negative impact on the diagnostic accuracy of IL-6 in patients undergoing hip and/or knee replacement.

## Supplementary Information

**Additional file 1: Table S1. **Search query of electronic databases.

**Additional file 2: Table S2. **Analysis of the subgroup.

## Data Availability

All data generated or analyzed during this study are included in this published article/as Additional files.
