# Supplementary material for: Serum versus synovial fluid interleukin-6 for periprosthetic joint infection diagnosis: a systematic review and meta-analysis of 30 diagnostic test accuracy studies
Source: J Orthop Surg Res. 2022 Dec 24;17:564. doi: 10.1186/s13018-022-03458-x (PMC9789601; doi:10.1186/s13018-022-03458-x)
Supplement: Supplementary file 1 — Additional file 1: Table S1. Search query of electronic databases. [file 13018_2022_3458_MOESM1_ESM.docx]

| **Search queries of Pubmed** | |
| --- | --- |
| Search | Query |
| 17 | #15 AND #16 |
| 16 | (diagnose) OR (diagnostic) |
| 15 | #4 AND #14 |
| 14 | joint infection*[Title/Abstract] OR #13 |
| 13 | #9 AND #12 |
| 12 | #10 OR #11 |
| 11 | Infection*[Title/Abstract] |
| 10 | Infections[MeSH Terms] |
| 9 | #5 OR #6 OR #7 OR #8 |
| 8 | Joint Prostheses[Title/Abstract] |
| 7 | Joint Prosthesis[MeSH Terms] |
| 6 | (((Joint Prosthesis Implantation*[Title/Abstract]) OR (Replacement Arthroplast*[Title/Abstract])) OR (Joint Replacement*[Title/Abstract])) OR (Total Joint Replacement*[Title/Abstract]) |
| 5 | Arthroplasty, Replacement[MeSH Terms] |
| 4 | #1 OR #2 OR #3 |
| 3 | (((Biomarkers[MeSH Terms]) OR (marker*[Title/Abstract])) OR (Biomarker*[Title/Abstract])) AND (inflammatory[Title/Abstract]) |
| 2 | (((((((((((((((((((Interleukin 6[Title/Abstract]) OR (IL6[Title/Abstract])) OR (IL-6[Title/Abstract])) OR (B-Cell Stimulatory Factor 2[Title/Abstract])) OR (B-Cell Stimulatory Factor-2[Title/Abstract])) OR (B Cell Stimulatory Factor-2[Title/Abstract])) OR (B Cell Stimulatory Factor 2[Title/Abstract])) OR (B-Cell Differentiation Factor[Title/Abstract])) OR (B Cell Differentiation Factor[Title/Abstract])) OR (BSF-2[Title/Abstract])) OR (Hybridoma Growth Factor[Title/Abstract])) OR (Plasmacytoma Growth Factor[Title/Abstract])) OR (Hepatocyte-Stimulating Factor[Title/Abstract])) OR (Hepatocyte Stimulating Factor[Title/Abstract])) OR (MGI-2[Title/Abstract])) OR (Myeloid Differentiation-Inducing Protein[Title/Abstract])) OR (Myeloid Differentiation Inducing Protein[Title/Abstract])) OR (Interferon beta-2[Title/Abstract])) OR (Interferon beta 2[Title/Abstract])) OR (IFN-beta 2[Title/Abstract]) |
| 1 | "Interleukin-6"[Mesh] |

| **Search queries of Cochrane library** | |
| --- | --- |
| Search | Query |
| 17 | #15 AND #16 |
| 16 | (diagnose) OR (diagnostic) |
| 15 | #4 AND #14 |
| 14 | joint infection*[Title/Abstract] OR #13 |
| 13 | #9 AND #12 |
| 12 | #10 OR #11 |
| 11 | Infection*[Title/Abstract] |
| 10 | Infections[MeSH Terms] |
| 9 | #5 OR #6 OR #7 OR #8 |
| 8 | Joint Prostheses[Title/Abstract] |
| 7 | Joint Prosthesis[MeSH Terms] |
| 6 | (((Joint Prosthesis implantation[Title/Abstract]) OR (Replacement Arthroplast*[Title/Abstract])) OR (Joint Replacement*[Title/Abstract])) OR (Total Joint Replacement*[Title/Abstract]) |
| 5 | Arthroplasty, Replacement[MeSH Terms] OR |
| 4 | #1 OR #2 OR #3 |
| 3 | (((Biomarkers[MeSH Terms]) OR (marker*[Title/Abstract])) OR (Biomarker*[Title/Abstract])) AND (inflammatory[Title/Abstract]) |
| 2 | (((((((((((((((((((Interleukin 6[Title/Abstract]) OR (IL6[Title/Abstract])) OR (IL-6[Title/Abstract])) OR (B-Cell Stimulatory Factor 2[Title/Abstract])) OR (B-Cell Stimulatory Factor-2[Title/Abstract])) OR (B Cell Stimulatory Factor-2[Title/Abstract])) OR (B Cell Stimulatory Factor 2[Title/Abstract])) OR (B-Cell Differentiation Factor[Title/Abstract])) OR (B Cell Differentiation Factor[Title/Abstract])) OR (BSF-2[Title/Abstract])) OR (Hybridoma Growth Factor[Title/Abstract])) OR (Plasmacytoma Growth Factor[Title/Abstract])) OR (Hepatocyte-Stimulating Factor[Title/Abstract])) OR (Hepatocyte Stimulating Factor[Title/Abstract])) OR (MGI-2[Title/Abstract])) OR (Myeloid Differentiation-Inducing Protein[Title/Abstract])) OR (Myeloid Differentiation Inducing Protein[Title/Abstract])) OR (Interferon beta-2[Title/Abstract])) OR (Interferon beta 2[Title/Abstract])) OR (IFN-beta 2[Title/Abstract]) |
| 1 | "Interleukin-6"[Mesh] |

| **Search queries of Embase** | |
| --- | --- |
| Search | Query |
| #23 | #21 AND #22 |
| #22 | diagnose OR diagnostic |
| #21 | #8 AND #20 |
| #20 | #17 OR #18 |
| #18 | 'joint infection*':ab,ti |
| #17 | #13 AND #16 |
| #16 | #14 OR #15 |
| #15 | 'autoinfection infection* infectious disease infectivity':ab,ti OR infection*:ab,ti OR 'infectious disease':ab,ti OR infectivity:ab,ti |
| #14 | 'infection'/exp |
| #13 | #9 OR #10 OR #11 OR #12 |
| #12 | 'arthroplasty prosthesis arthroprosthesis articulated prosthesis':ab,ti OR arthroprosthesis:ab,ti OR 'articulated prosthesis':ab,ti OR 'artificial joint hemicap joint endoprosthesis joint implant':ab,ti OR hemicap:ab,ti OR 'joint endoprosthesis':ab,ti OR 'joint implant':ab,ti OR ('joint prostheses joint total prosthesis orthosphere':ab,ti AND device:ab,ti AND 'prosthetic joint total joint prosthesis':ab,ti) OR 'joint total prosthesis':ab,ti OR (orthosphere:ab,ti AND device:ab,ti) OR 'prosthetic joint':ab,ti OR 'total joint prosthesis':ab,ti |
| #11 | 'joint prosthesis'/exp |
| #10 | 'joint replacement':ab,ti |
| #9 | 'replacement arthroplasty'/exp |
| #8 | #1 OR #2 OR #7 |
| #7 | #5 AND #6 |
| #6 | inflammatory:ab,ti |
| #5 | #3 OR #4 |
| #4 | biomarker*:ab,ti OR marker*:ab,ti |
| #3 | 'biological marker'/exp |
| #2 | b cell stimulating factor 2':ab,ti OR '26 k protein':ab,ti OR 'b cell stimulatory factor 2':ab,ti OR 'b lymphocyte stimulating factor 2':ab,ti OR 'beta 2 interferon':ab,ti OR 'beta2 interferon':ab,ti OR 'bsf 2 bsf2':ab,ti OR bsf2:ab,ti OR 'hepatocyte stimulating factor il 6':ab,ti OR 'il 6':ab,ti OR 'interferon beta 2 interferon beta2 interleukin b':ab,ti OR 'interferon beta2':ab,ti OR 'interleukin b':ab,ti OR 'interleukin hp1 interleukin-6 liver cell stimulating factor plasmacytoma growth factor protein 26k':ab,ti OR 'interleukin 6':ab,ti OR 'liver cell stimulating factor':ab,ti OR 'plasmacytoma growth factor':ab,ti OR 'protein 26k':ab,ti |
| #1 | 'interleukin 6'/exp |
