# Supplementary material for: Serum versus synovial fluid interleukin-6 for periprosthetic joint infection diagnosis: a systematic review and meta-analysis of 30 diagnostic test accuracy studies
Source: J Orthop Surg Res. 2022 Dec 24;17:564. doi: 10.1186/s13018-022-03458-x (PMC9789601; doi:10.1186/s13018-022-03458-x)
Supplement: Supplementary file 2 — Additional file 2: Table S2. Analysis of the subgroup. [file 13018_2022_3458_MOESM2_ESM.docx]

Table S2. Subgroup analysis

| Source | Number of trails | Heterogeneity  (Sen/Spe) | Sensitivity (95% CI) | Specificity (95% CI) | AUC (95% CI) | PLR (95% CI) | NLR (95% CI） | DOR (95% CI) |
| --- | --- | --- | --- | --- | --- | --- | --- | --- |
| **Serum IL-6** | | | | | | | | |
| Overall | 23 | 72.62%/75.47% | 0.76 (0.69, 0.81) | 0.88 (0.82, 0.92) | 0.88 (0.85, 0.91) | 6.2 (4.3, 9.0) | 0.28 (0.22, 0.35) | 22 (14, 36) |
| Exclusion of inflammatory disease | | | | | | | | |
| Yes, and NA | 18 | 75.79%/78.40% | 0.78 (0.71, 0.84) | 0.87 (0.80, 0.92) | 0.89 (0.86, 0.91) | 6.2 (4.0, 9.7) | 0.25 (0.19, 0.33) | 25 (14, 44) |
| No | 5 | 58.54%/51.69% | 0.68 (0.57, 0.77) | 0.89 (0.81, 0.94) | 0.85 (0.82, 0.88) | 6.0 (3.5, 10.6) | 0.36 (0.27, 0.49) | 17 (8, 35) |
| Mixed parts | 20 | 74.18%/78.26% | 0.78 (0.71, 0.83) | 0.88 (0.82, 0.93) | 0.88 (0.85, 0.91) | 6.66 (4.2, 10.4) | 0.25 (0.20, 0.33) | 26 (15, 45) |
| MSIS criteria | 16 | 76.34%/72.45% | 0.74 (0.66, 0.80) | 0.85 (0.79, 0.89) | 0.85 (0.79, 0.89) | 4.9 (3.6, 6.8) | 0.31 (0.24, 0.39) | 16 (10, 25) |
| Study design | | | | | | | | |
| prospective | 15 | 59.10%/82.29% | 0.77 (0.71, 0.82) | 0.90 (0.81, 0.95) | 0.86 (0.83, 0.89) | 7.7 (3.9, 15.1) | 0.26 (0.20, 0.33) | 30 (13, 68) |
| retrospective | 6 | 86.42%/35.15% | 0.71 (0.56, 0.83) | 0.87 (0.81, 0.90) | 0.88 (0.81, 0.91) | 5.3 (4.1, 6.8) | 0.33 (0.21, 0.52) | 16 (9, 26) |
| Number of patients | | | | | | | | |
| ≥60 | 15 | 78.43%/75.05% | 0.74 (0.67, 0.81) | 0.85 (0.80, 0.90) | 0.87 (0.84, 0.90) | 5.1 (3.7, 7.0) | 0.30 (0.23, 0.39) | 17 (11, 26) |
| < 60 | 8 | 50.90%/79.56% | 0.79 (0.66, 0.88) | 0.94 (0.80, 0.98) | 0.90 (0.80, 0.92) | 13.3 (3.6, 48.9) | 0.22 (0.13, 0.37) | 60 (13, 264) |
| Cut-off | | | | | | | | |
| ≥10 pg/ml | 8 | 21.02%/79.60% | 0.80 (0.72, 0.86) | 0.95 (0.82, 0099) | 0.85 (0.82, 0.88) | 15.8 (4.2, 59.5) | 0.21 (0.15, 0.30) | 74 (17, 321) |
| <10 pg/ml | 14 | 78.67%/72.63% | 0.73 (0.65, 0.80) | 0.84 (0.78, 0.89) | 0.86 (0.83, 0.89) | 4.5 (3.3, 6.2) | 0.32 (0.25, 0.42) | 14 (9, 21) |
| **synovial fluid IL-6** | | | | | | | | |
| Overall | 14 | 92.81%/80.14% | 0.87 (0.75, 0.93) | 0.90 (0.85, 0.93) | 0.94 (0.92, 0.96) | 8.5 (5.3, 13.6) | 0.15 (0.08, 0.29) | 57 (21, 156) |
| Inclusion of inflammatory disease | | | | | | | | |
| Yes and NA | 4 | NA | NA | NA | NA | NA | NA | NA |
| No | 10 | 93.98%/83.48% | 0.85 (0.67, 0.93) | 0.91 (0.84, 0.95) | 0.94 (0.92, 0.96) | 8.9 (4.9, 16.3) | 0.17 (0.07, 0.40) | 52 (14, 191) |
| Mixed parts | 13 | 93.68%/80.39% | 0.88 (0.76, 0.94) | 0.90 (0.84, 0.93) | 0.94 (0.92, 0.96) | 8.4 (5.1, 13.9) | 0.14 (0.07, 0.28) | 61 (20, 184) |
| MSIS criteria | 10 | 94.43%/78.78% | 0.86 (0.72, 0.94) | 0.88 (0.82, 0.93) | 0.93 (0.91, 0.95) | 7.4 (4.5, 12.3) | 0.16 (0.07, 0.35) | 47 (15, 147) |
| Study design | | | | | | | | |
| prospective | 11 | 94.23%/62.36% | 0.87 (0.72, 0.95) | 0.90 (0.84, 0.94) | 0.95 (0.92, 0.96) | 9.0 (5.0, 15.9) | 0.14 (0.06, 0.34) | 63 (17, 232) |
| retrospective | 2 | NA | NA | NA | NA | NA | NA | NA |
| Number of patients | | | | | | | | |
| ≥80 | 8 | 43.60%/80.34% | 0.85 (0.80, 0.89) | 0.89 (0.82, 0.93) | 0.90 (0.87, 0.93) | 7.7 (4.6, 13.0) | 0.16 (0.12, 0.23) | 47 (22, 101) |
| < 80 | 6 | 96.20%/79.75% | 0.89 (0.52, 0.98) | 0.91 (0.82, 0.96) | 0.95 (0.92, 0.96) | 10.3 (4.3, 24.7) | 0.12 (0.02, 0.73) | 85 (7, 1017) |
| Cut-off | | | | | | | | |
| ≥2300 pg/ml | 6 | 38.79%/83.49% | 0.87 (0.80, 0.92) | 0.90 (0.84, 0.94) | 0.93 (0.90, 0.95) | 9.0 (5.4, 14.9) | 0.14 (0.10, 0.22) | 62 (33, 117) |
| < 2300 pg/ml | 7 | 96.14%/85.40% | 0.88 (0.60, 0.97) | 0.91 (0.81, 0.96) | 0.95 (0.93, 0.97) | 9.7 (3.9, 24.0) | 0.14 (0.03, 0.56) | 72 (8, 612) |

Sen, sensitivity; Spe, specificity; AUC, area under the curve; PLR, positive likelihood ratio; NLR, negative likelihood ratio; DOR, diagnostic odds ratio; NA, not available.
